# Contribution to Establishing a Foundation for Promoting Cancer Control in Japan by Providing Precise Cancer Information and Establishing a Research Infrastructure―Secondary Publication

**DOI:** 10.31662/jmaj.2024-0379

**Published:** 2025-04-04

**Authors:** Tomotaka Sobue

**Affiliations:** 1 Osaka University, Osaka, Japan

**Keywords:** cancer statistics, cancer registry, cancer screening, cancer control

## Abstract

At the beginning of 2000, the National Cancer Center had little involvement in cancer statistics. In addition, cancer incidence data were collected by regional cancer registries (approximately 30 prefectures have on-site registries), but the problems were that (1) the completeness of the registration was low, (2) the registration method was not standardized, and (3) usage was limited. In the Third Comprehensive Strategic Cancer Research Project (2004-2013), Field 7, “Research on Developing Cancer Surveillance System and Disseminating Cancer Information,” was added, and we could build a system to aggregate the latest cancer statistical data from Japan and provide it as content for cancer information services. In addition, within the Regional Cancer Registry Research Group, we promoted the standardization of population-based cancer registry methods through “formulation and dissemination of standard registration forms” and “development and dissemination of standard registry systems.” We believe that these factors served as the basis for the smooth transfer to the National Cancer Registry under the Cancer Registry Promotion Act, which was enacted in 2013.

In contrast, to take over the function of Hisamichi’s “Evaluation of the Effectiveness of New Cancer Screening Methods” report published in 2001, the Ministry of Health, Labour and Welfare Cancer Research Grant “Research on the Establishment of Appropriate Methods for Cancer Screening and Evaluation Methods” group, which started in 2003, formulated the guideline development procedure and updated the guidelines, and the work of updating the guidelines was subsequently taken over by the National Cancer Center. This remains an important mechanism to ensure that cancer screening methods that do not have a scientifically confirmed balance between benefits and harms are not introduced into policy.

## I. Introduction

**Figure 1.**
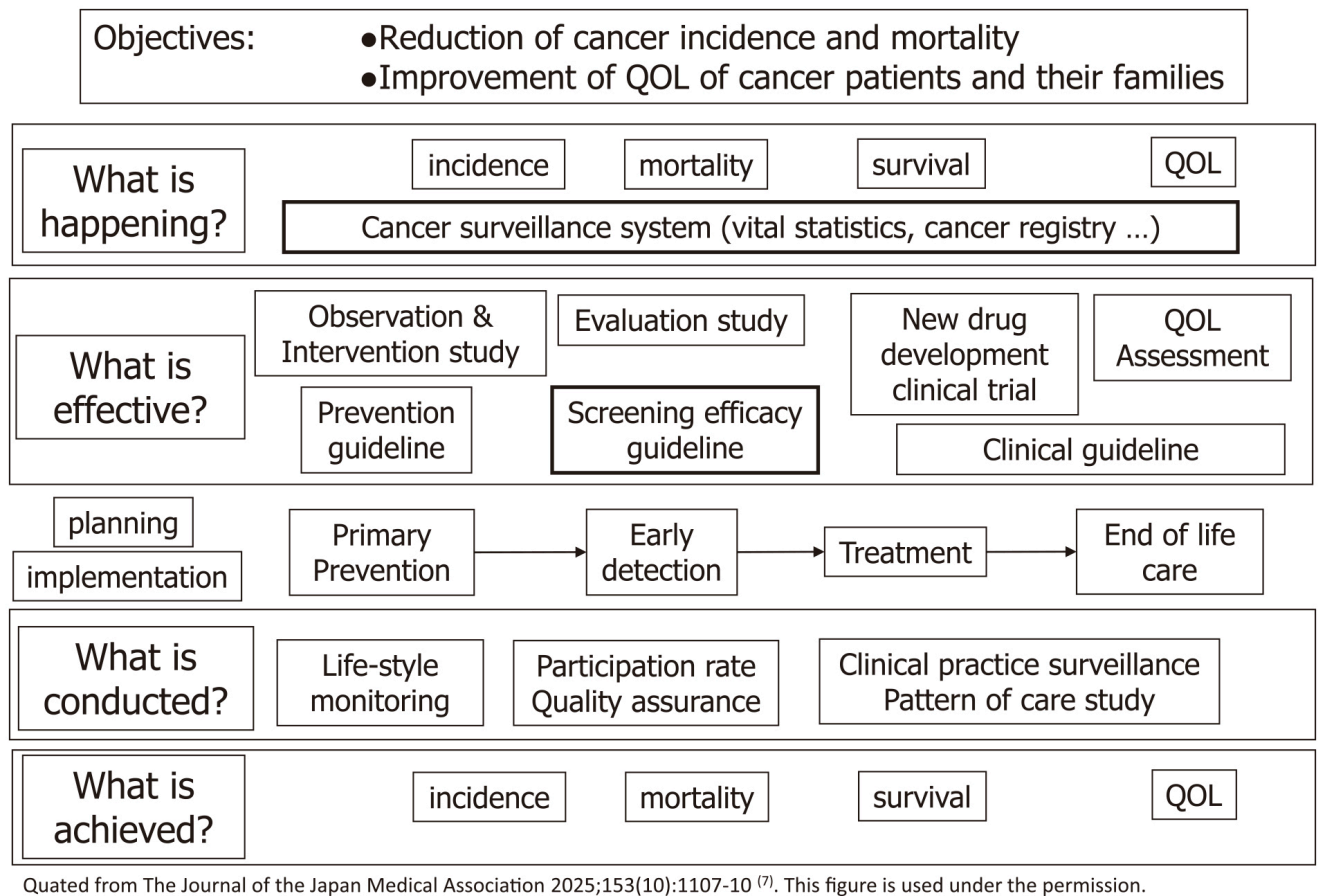
Steps for implementing data-based cancer control program.

The purpose of cancer control is to reduce cancer incidence and mortality and improve the quality of life of patients with cancer and their families. To achieve this goal efficiently, the implementation of cancer control must be based on data. Data are needed not only about the “actual state of cancer” and “effective means of reducing cancer incidence and mortality and improving the quality of life of cancer patients and their families” but also about “whether the measures are being implemented correctly” (Figure 1). The reasons for this award, “Compilation of cancer statistics (mortality and incidence) information” and “Standardization of regional cancer registration,” are activities related to the system for grasping the “actual state of cancer,” whereas the “Guidelines for the effectiveness of cancer screening” are activities related to the system for determining “effective means of reducing cancer deaths regarding cancer screening.” This article provides an overview of these activities.

## II. Establishment of Cancer Statistics (Mortality and Incidence) Information

Currently, when searching for information on cancer statistics, it is possible to obtain the latest and most accurate information by accessing the Cancer Statistics section of the National Cancer Center’s Cancer Information Service ^(1)^. In 2002, when the author became Director of the Cancer Information Research Department at the National Cancer Center Research Institute, the National Cancer Center had almost no involvement in activities related to domestic cancer statistics. The only activity in which the National Cancer Center was involved was editing the booklet titled “Cancer Statistics,” published by the Cancer Research Promotion Foundation, but even in that process, mortality statistics were provided by the Statistics and Information Department of the Ministry of Health, Labour and Welfare, and incidence data were provided by the Osaka Medical Center for Cancer and Cardiovascular Diseases. A summary of cancer statistics, “Cancer Statistics White Paper” (Shinohara Publishing), was published in 1993, 1999, and 2004, but the authors were staff members of the Aichi Cancer Center and the Osaka Medical Center for Cancer and Cardiovascular Diseases, and there was no involvement of the National Cancer Center.

The First and Second Comprehensive Strategic Cancer Research Project mainly aimed to strengthen basic research to combat cancer. In the Third Comprehensive Strategic Cancer Research Project that began in 2004, a new area, “Research on developing cancer surveillance system and disseminating cancer information,” was added as area 7, and the author was the principal investigator. As part of this, cancer mortality and cancer incidence data were compiled and collected at the National Cancer Center, and cancer statistical data began to be provided as content for the cancer information service. To this day, these data are widely used not only by experts but also by the public, including in the media and in education.

## III. Standardization of Regional Cancer Registries

**Figure 2.**
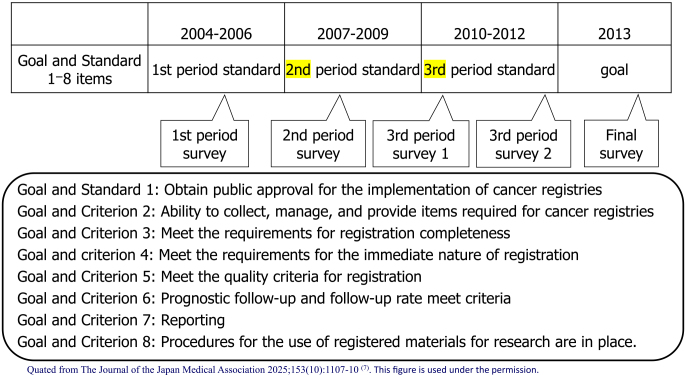
Time schedule of the cancer registry research group in the 3rd Comprehensive Cancer Strategy Research.

**Figure 3.**
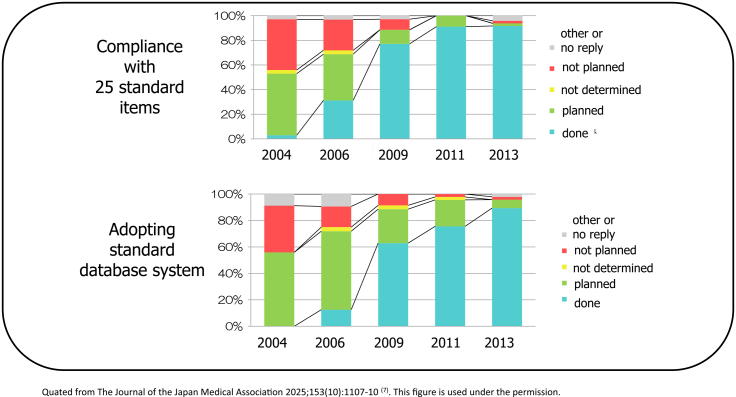
Trends of standardization index for regional cancer registry.

In 2000, Japan’s incidence data were collected through regional cancer registries (approximately 30 prefectures have on-site registries) run by prefectures, but there were problems in that (1) the registration completeness was low; (2) the registration method was not standardized, and (3) the data were not used ^(2)^. In the Third Comprehensive Cancer Strategy Research Area 7, the research group titled “Research on the surveillance system for cancer incidence and mortality trends” (Chief Researcher: Tomotaka Sobue) divided the 10-year period from 2004 to 2013 into three periods to address the issue of “(2) the registration method was not standardized.” The research group set achievement standards and final goals for promoting standardization in eight areas (public approval, registration items, completeness, immediacy, quality, prognosis surveys, reports, and research use) and periodically checked the progress of achievement (Figure 2). In particular, standardization was promoted through “establishment and dissemination of standard registration forms” and “development and dissemination of standard registration systems.” As a result, although the percentage of those who “adapted to the standard registration form” and “adopted the standard registration system” was almost 0% at the start of 2004, this figure reached almost 100% by the end of 2013, and standardization has progressed dramatically (Figure 3). The factors behind this include (1) the National Cancer Center taking the lead, (2) the involvement of core members among prefectural staff to advance standardization, (3) sufficient research funding, and (4) the establishment of the Basic Act on Cancer Control, which clarified the position of population-based cancer registries. In contrast, it is important to always keep in mind that the pain is greater in prefectures that have been implementing high-quality cancer registries for a long time, as a factor hindering standardization ^(3)^.

**Figure 4.**
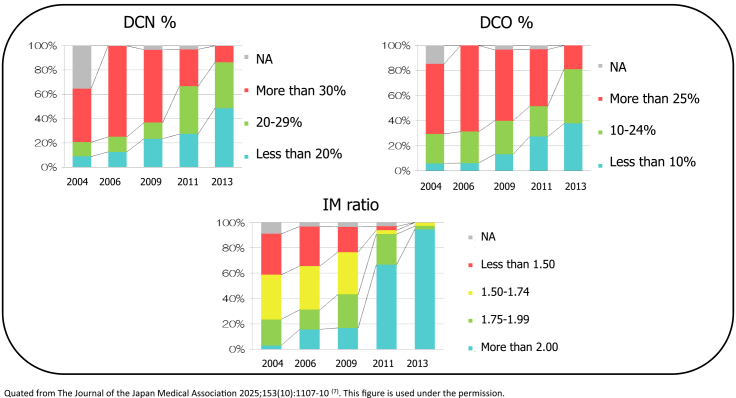
Trends of quality index for regional cancer registry.

During this time, there was also a significant improvement in the issue of “(1) low registration completeness” (Figure 4). This is not the result of the research group’s activities, but rather due to (1) the establishment of hospital-based cancer registries at designated cancer hospitals, which used to have very poor involvement in population-based cancer registries, and (2) the addition of cooperation with population-based cancer registries to the foundation for medical insurance claims.

The prefecture-wide regional cancer registries were transferred to a national project as a national cancer registry with registration obligations owing to the Cancer Registration Promotion Act enacted in 2013. Registration operations began with cases in 2016, and we believe that the standardization that had been under way up to that point contributed to a smooth start.

## IV. Guidelines for the Effectiveness of Cancer Screening

The national cancer control program of Japan began with cancer screening, which was introduced as part of the health care program for older adults based on the Health and Medical Care for the Elderly Act (1982). Stomach and uterine cancer screening was introduced in 1983, lung and breast cancer screening in 1987, and colon cancer screening in 1992. However, except for colon cancer screening, these were not introduced as policies after the effectiveness of reducing deaths from the cancers in question was confirmed. At the time, it was recognized domestically that the effectiveness of screening was confirmed by the improvement in survival rates for cancers detected by screening, but internationally, it is known that evaluation based on survival rates overestimates the effectiveness owing to bias, and the effect of reducing mortality was essential to confirm the effectiveness. Thereafter, evaluation studies with cancer mortality rates as the end point were conducted domestically, mainly case-control studies, and in 2001, the report “Evaluation of the Effectiveness of New Cancer Screening Methods” by Professor Hisamichi’s group ^(4)^ found that stomach, cervical, lung, breast, and colon cancer screening had an effect of reducing mortality, and confusion regarding the issue of effectiveness was resolved.

**Figure 5.**
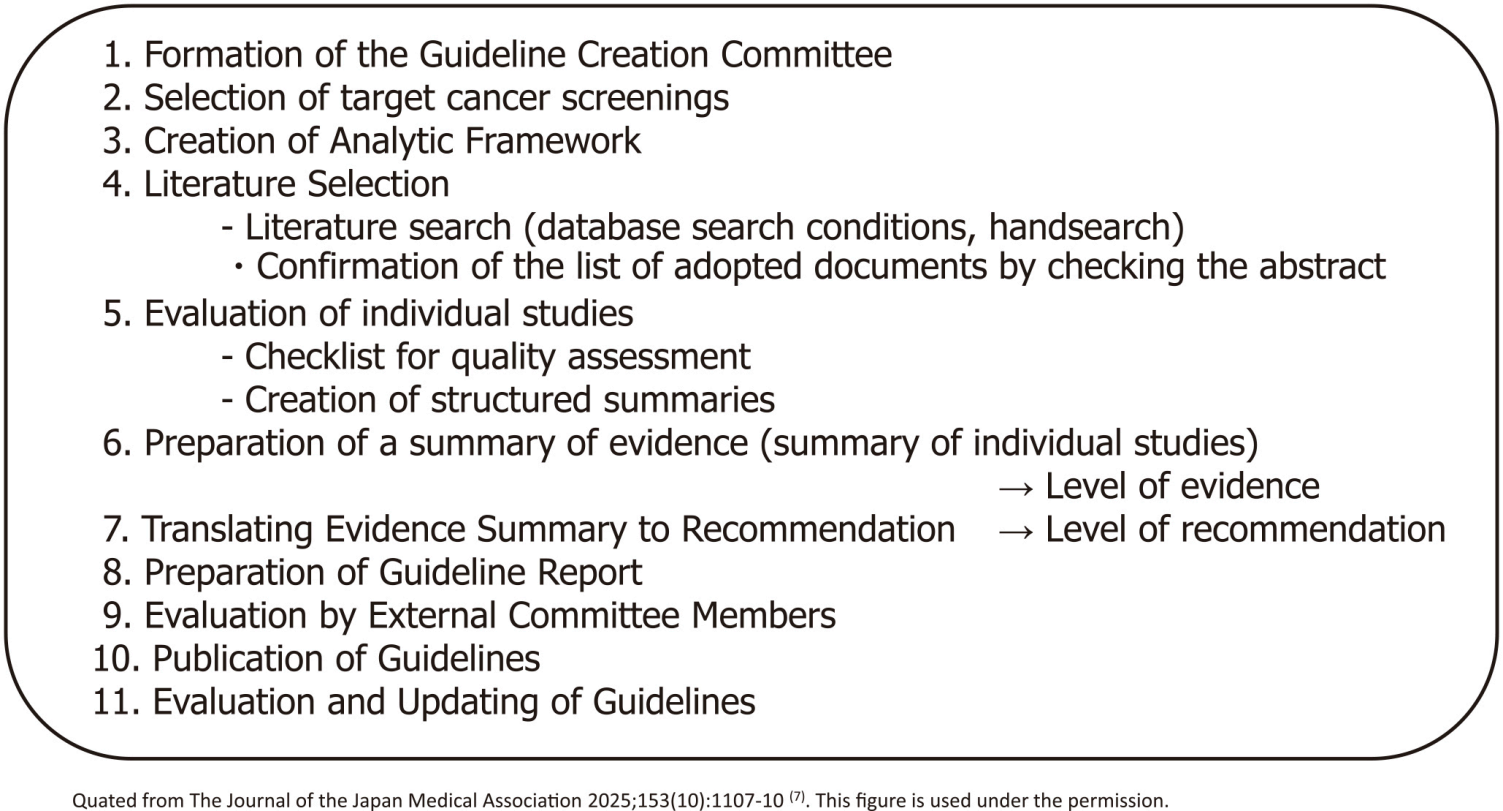
Procedure for creating cancer screening efficacy evaluation guidelines.

**Figure 6.**
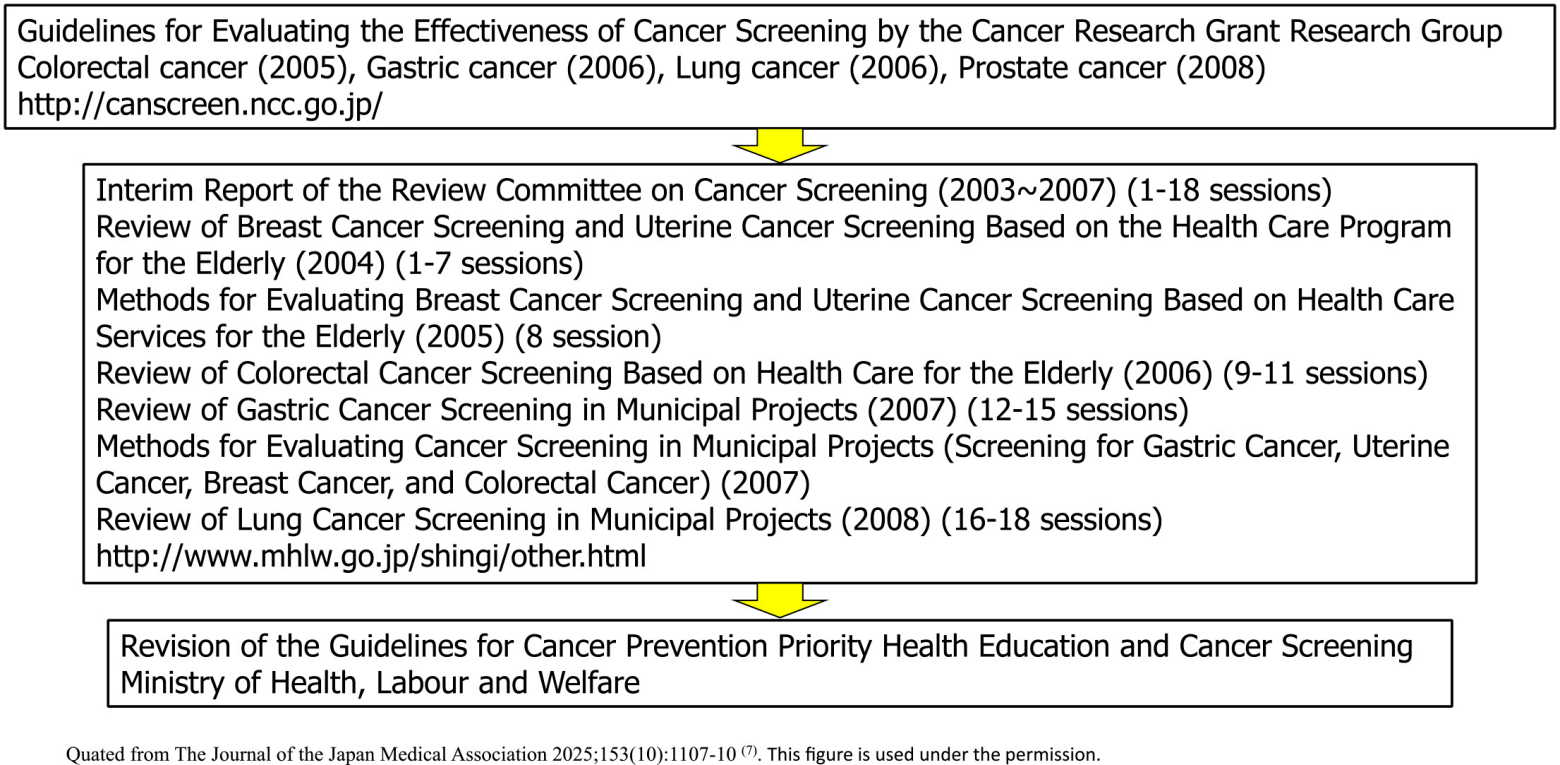
Process of policy introduction of screening methods that have been shown to be effective based on scientific evidences.

In other countries, public institutions have established a system for regularly updating “Guidelines for the Evaluation of the Effectiveness of Cancer Screening (documents that conduct systematic reviews and determine the recommendation grade)” (such as the US Preventive Services Task Force), and it was necessary also to establish a system for updating the Hisamichi group’s report in Japan. In 2003, the Ministry of Health, Labour and Welfare Cancer’s Research Grant “Research on the Establishment of Appropriate Methods for Cancer Screening and Its Evaluation Methods” research group (Chief Researcher: Tomotaka Sobue) was launched, and a common guideline development procedure was formulated consulting globally recognized documents, such as the US Preventive Services Task Force (Figure 5). At that time, an analytic framework was adopted to use not only direct evidence of the effect of reducing mortality but also indirect evidence, and conditions for determining the level of evidence and the recommendation level were determined ^(5)^. Based on this, guidelines were updated for colorectal, stomach, lung, prostate, and cervical cancer ^(6)^. This initiative was then taken over by the National Cancer Center Research and Development Grant Research Team (Chief Researcher: Hiroshi Saito and Tomio Nakayama) and continues to this day. Furthermore, when the balance between benefits and harms of a new screening method is confirmed and the guideline grade is updated to a level that recommends its introduction into policy, the “Implementation Guidelines for Cancer Screening” are revised after deliberation at the review committee of the Ministry of Health, Labour and Welfare, and the path to policy introduction is established (Figure 6). This remains an important mechanism to ensure that cancer screening methods whose balance between benefits and harms has not been scientifically confirmed are not introduced into policy.

## Article Information

This article is based on the study, which received the Medical Award of The Japan Medical Association in 2024. This is a revised English version of the article originally published in Japanese in the Journal of the Japan Medical Association 2025;153(10):1107-10 ^(7)^. The original version is available at https://med.or.jp/cme/jjma/newmag/pdf/153101107.pdf. Only members of the Japan Medical Association are able to access it.

The Editors-in-Chief of the Journal of the Japan Medical Association and JMA Journal have permitted the publication of this manuscript.

### Conflicts of Interest

None

## Acknowledgement

I express my deep gratitude to my mentors, Dr. Isaburo Fujimoto, Dr. Setsuo Hirohashi, and Dr. Shigeru Hisamichi, in addition to the staff of the Center for Cancer Control and Information Services, National Cancer Center (now the Institute of Cancer Control, National Cancer Center). I also thank the Japan Epidemiological Association for recommending me for this award.
